# The Simultaneous Assessment of Time and Motion Response during Dual Tasks

**DOI:** 10.3390/s23115309

**Published:** 2023-06-03

**Authors:** Marrone Flavia, Donno Lucia, Lopreside Antonia, Piccinini Luigi, Tarabini Marco, Galli Manuela

**Affiliations:** 1 Department of Mechanical Engineering, Politecnico di Milano, via Privata Giuseppe La Masa 1, 20156 Milan, Italy; flavia.marrone@polimi.it (M.F.); marco.tarabini@polimi.it (T.M.); 2 Department of Electronics, Information and Bioengineering, Politecnico di Milano, Piazza Leonardo da Vinci 32, 20133 Milan, Italy; lucia.donno@polimi.it; 3 School of Industrial and Information Engineering, Politecnico di Milano, Piazza Leonardo da Vinci 32, 20133 Milan, Italy; antonia.lopreside@mail.polimi.it; 4 Scientific Institute, IRCCS Eugenio Medea, Bosisio Parini, 23842 Lecco, Italy; luigi.piccinini@lanostrafamiglia.it

**Keywords:** reaction time, motor response, proximity sensor, light stimulus, motion analysis, dual task

## Abstract

Measurement of reaction time in clinical settings is generally employed to assess cognitive abilities by having a subject perform standard simple tests. In this study, a new method of measuring response time (RT) was developed using a system composed of LEDs that emit light stimuli and are equipped with proximity sensors. The RT is measured as the time taken by the subject to turn off the LED target by moving the hand towards the sensor. Through an optoelectronic passive marker system, the associated motion response is assessed. Two tasks of 10 stimuli each were defined: simple reaction time and recognition reaction time tasks. To validate the method implemented to measure RTs, the reproducibility and repeatability of the measurements were estimated, and, to test the method’s applicability, a pilot study was conducted on 10 healthy subjects (6 females and 4 males, age = 25 ± 2 years), reporting, as expected, that the response time was affected by the task’s difficulty. Unlike commonly used tests, the developed method has proven to be adequate for the simultaneous evaluation of the response in terms of time and motion. Furthermore, thanks to the playful nature of the tests, this method could also be used for clinical and pediatric applications to measure the impact of motor and cognitive impairments on RT.

## 1. Introduction

The ability to elicit a goal-directed motor response to visual stimuli is a key aspect of our daily life activities: an individual with a short reaction time will be able to respond quickly to surrounding events. However, it could be compromised in subjects with motor and/or cognitive impairments. Specifically, the time taken to perform a task could be affected by different conditions, i.e., old age or the presence of pathologies [1,2,3,4]. Specifically in the pediatric population, different studies have investigated the impact of deficits in cognitive abilities. In children with Down syndrome (DS), motor and cognitive development is delayed with respect to typically developed ones [5]. In the case of autistic spectrum disorders (ASD), there may be difficulties in shifting visual attention [6,7]. As regards hemiplegic cerebral palsy (HCP), the critical role of vision for efficient planning and performing tasks was demonstrated by Surkar et al. [8], highlighting the need for therapeutic interventions focused on improving visuomotor coordination to enhance motor performance.

In clinical settings, clinical scales or standard simple tests are commonly used to evaluate motor and cognitive abilities [9,10,11].

In particular, an efficient parameter used to assess the subject’s moto-cognitive ability, as evidenced in [12], is “reaction time”, defined as the time elapsing between the start of the stimulus and the beginning of the subject’s reaction. Specifically, three test paradigms could be applied: (1) in “simple reaction time” tests, a single stimulus is applied and a single response is expected; (2) in “choice reaction time” experiments, random stimuli are applied and a specific response for each stimulus is expected [13,14]; (3) in “recognition reaction time” tests, there are both stimuli that should get a response and a “distractors set” that should not be responded to.

Reaction time tests are widely used in pediatric clinical settings to assess cognitive abilities in the case of several diseases by comparing the test results of pathological children with a control group by using digital tests or devices dedicated to stimulus generation and reaction time recording.

A computerized system used in the pediatric field is the Cambridge Neuropsychological Test Automated Battery (CANTAB) (Cambridge Cognition Ltd., Cambridge, United Kingdom) [15]. It is software that allows the execution of various cognitive tests in which the subject responds to visual stimuli by interacting with a touchscreen or keyboard. With analogous functionalities, there is also the Vienna Test System (Schuhfried, Mödling, Austria) that provides digital tests with the use of a computer and a hand controller [16].

Regarding reaction time recording with dedicated up-to-date instrumentation, Dik et al. [17] assessed the visuomotor reaction time (VMRT) in children with respiratory diseases with a Fitlight Trainer (FITLIGHT Sports Corp., Aurora, ON, Canada), based on six light-emitting diode sensors. Children were positioned in front of the sensor set and the total time to touch all the luminous sensors was recorded as their VMRT. Several technological systems have been proposed in the past few years, with the purpose of accurately measuring RT while avoiding overestimation due to hardware and software delays in computer-based paradigms [18]. The D2 Visuomotor Training Device (Dynavision™, Cincinnati, Ohio, USA) emerged as a reliable device for assessing reaction time to both central and peripheral visual stimuli, able to separately quantify visual from motor reaction time [19]. Furtherly, NeuroTracker (NeuroTracker, Montreal, QC, Canada), was developed to improve cognitive performance and ensure a real-world experience at the same time: targets are 3-D balls that are displayed and fluctuate around the screen, while a motion sensor monitors movements and converts them into a digital response [20].

Although all the mentioned methods are applied in practice or in clinical studies, they have some limitations. The clinical scales are operator-dependent and not sensitive to small variations, given the limited score range. Besides, an important aspect that should not be underestimated in the case of pediatric applications is the playful and inclusive nature of the tests. In this sense, the use of dedicated technologies is suitable, and more than the digital tests. Nevertheless, in addition to reaction time assessment, no motion analysis is carried out. The quantification of joint kinematics during the motor response would be useful from different perspectives in clinical settings: it would help to achieve a more comprehensive understanding of the subject’s response, to reveal the compensatory strategies underlying the recorded motor reaction time, and to monitor in-depth motor improvements, for example, after specific therapy. This information cannot be easily derived from the measurement of response time alone; hence, in our opinion, it would be useful to integrate it with the quantification of kinematics. To the best of our knowledge, only one study combined the passive marker-based motion analysis with the quantification of the response time [8]. However, the authors used a motion capture system during the test to record just the upper limb movements.

Considering all these aspects, an innovative and versatile protocol, combining a commercial device for measuring the cognitive response with a motion capture system, is proposed in this pilot study, allowing us to: (i) accurately quantify the subject’s response to visual stimuli, (ii) evaluate different subintervals of time in response, and (iii) quantify total-body kinematics while performing tasks of different difficulty levels.

## 2. Materials and Methods

### 2.1. Protocol Development

In this work, we developed a protocol for the simultaneous evaluation of response times and movement kinematics following a light stimulus using a motion capture system (SMART 400 DX—BTS Bioengineering, Garbagnate Milanese, Milan, Italy) composed of 6 infrared cameras (accuracy: <0.3 mm on a volume of 4 × 3 × 3 m; acquisition frequency: 100 Hz). The stimuli were emitted according to a predetermined pattern by WittySEM (Microgate©, Bolzano, Italy), a system typically used to train and measure athletes’ reaction time to visual stimuli, composed of 8 semaphores, which are 5 × 7 LEDs matrices, wirelessly connected to a chronometer (time resolution: 4 × 10^−5^ s; impulse transmission accuracy: ±0.4 ms). Each matrix is equipped with a sensor that detects the proximity of the hand when it is close to turning off the LED target.

**Figure 1:**
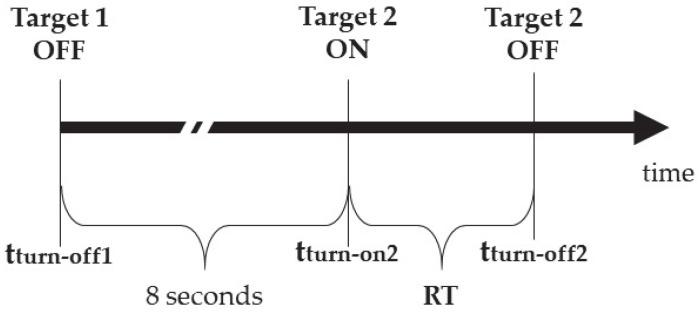
The temporal scheme for the calculation of RT.

Response time (hereinafter RT) is defined as the time between the appearance of the stimulus and the turn-off of the target, and it is measured as follows:

(1) $$\mathrm{RT}=\left(t_{\mathrm{turn}-\mathrm{off}\;2}-t_{\mathrm{turn}-\mathrm{off}\;1}\right)-\left(t_{\mathrm{turn}-\mathrm{on}\;2}-t_{\mathrm{turn}-\mathrm{off}\;1}\right)$$

where the interval between the time of turn-off ($t_{\mathrm{turn}-\mathrm{off}})$ of one stimulus and the time of turn-on ($t_{\mathrm{turn}-\mathrm{on}})$ of the following one is set at 8 s (Figure 1).

The semaphores are placed on a grid similar to those realized in previous studies with analogous systems, i.e., the Fitlight Trainer [17] or D2-Dynavision [19]. The subject is asked to stand in front of a grid and to perform different tasks, in which the target changes its position following a predetermined sequence unknown to the subject.

**Figure 2:**
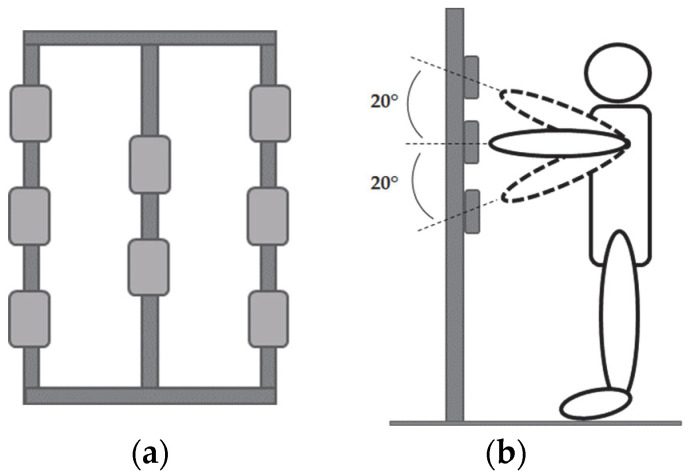
(**a**) Semaphore position on the grid; (**b**) subject position in front of the grid.

The grid is composed of three poles spaced at 30 cm: three semaphores are located on each of the lateral poles and two in the middle one. To ensure the same execution conditions for all subjects, some distances have been established: the distance between the grid and the subject is equal to the upper limb length, and the position of the top and bottom semaphores is such that there is a 20° angle between the extended upper limb and the horizontal line (Figure 2). This angle was deemed the most appropriate to reach the target without inducing difficulties.

**Figure 3:**
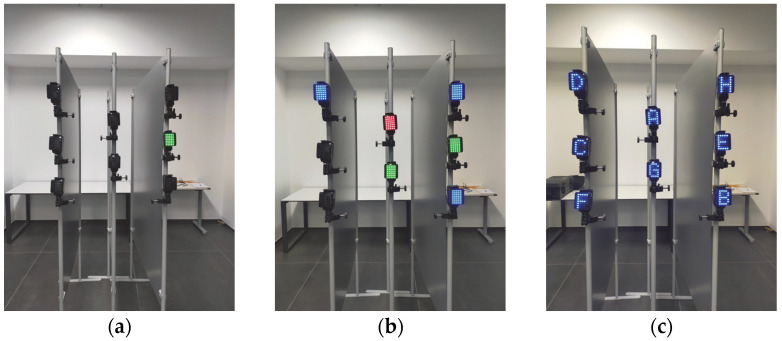
(**a**) SRT; (**b**) RRT-COL; (**c**) RRT-LET tasks.

Two tasks were considered (Figure 3):

• Simple response time (SRT): for each stimulus, only the target lights up. In this case, the subject is asked to perform the task separately with the right and left upper limbs (hereinafter SRT-R and SRT-L, respectively).
• Recognition response time (RRT): for each stimulus, other LEDs light up besides the target, and the subject freely chooses the upper limb to be used.
 For the RRT task, two levels of difficulty are provided:
  - RRT with colour distraction (hereinafter RRT-COL), in which, for each stimulus, other semaphores of different colours are lit together, but only one, of a specific colour, is the target;
  - RRT with symbol distraction (hereinafter RRT-LET), in which, for each stimulus, all matrices are simultaneously illuminated with the same colour, but with different symbols, that is, letters of the alphabet, one of which is the target.

Each task involves 10 stimuli. The first one is considered as the test onset, so each task has 9 measured RTs. Considering that it is not necessary to touch the target but only to reach the threshold for turn-off, when the subject reached for the target, but not close enough, thus recording RTs longer than the real ones, the stimuli were classified as outliers and neglected in the analyses.

**Figure 4:**
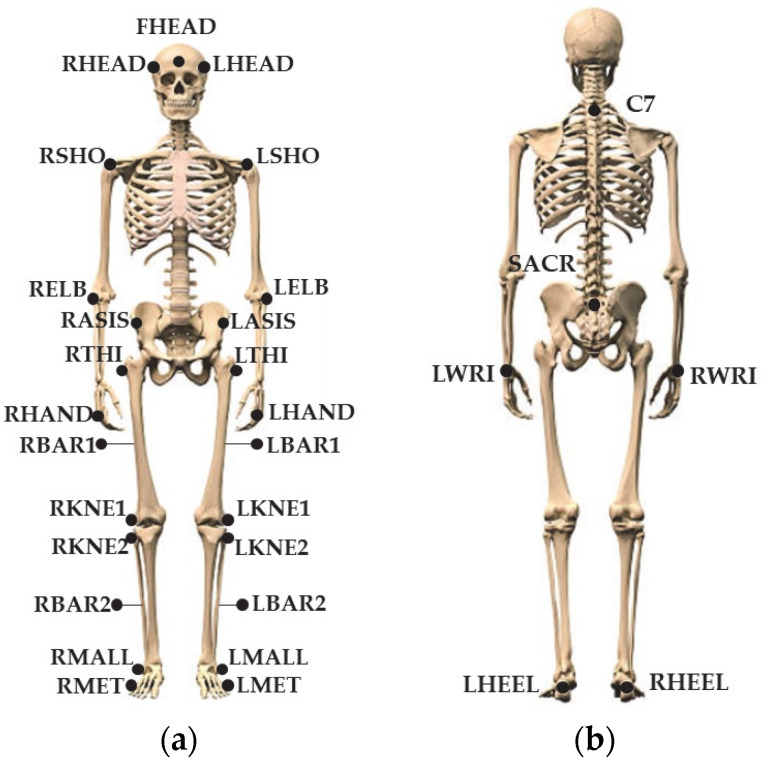
Marker positions: (**a**) front view of the subject; (**b**) back of the subject.

To evaluate RT and the motion response, we developed a protocol based on the motion capture of passive markers following the Davis model [21], with the addition of markers on the head (one marker on the front of the head and two on the temporal areas), upper limbs (one on the lateral epicondyle of the elbow and one on the ulnar styloid), and hands (one marker corresponding to the third carpometacarpal joint), as shown in Figure 4. To identify the semaphore position, six markers are also placed on the grid.

The RT is calculated considering that the instant of turn-off recorded by the proximity sensor coincides with the instant in which the minimum distance between the target and the hand’s marker was detected by the optoelectronic system.

**Figure 5:**
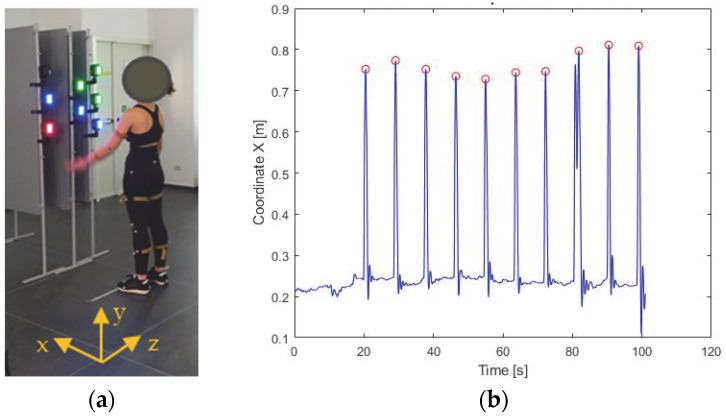
Subject approaching the hand to the target (**a**). The x-coordinate of the hand’s marker over time along the anterior-posterior axis of the absolute reference system. The red dots indicate the target’s turn-off instants (**b**).

Figure 5a shows the subject approaching the hand to the target. Figure 5b shows the hand displacement along the anterior-posterior axis; peak values, i.e., when the target is turned off, are shown by a red dot.

**Figure 6:**
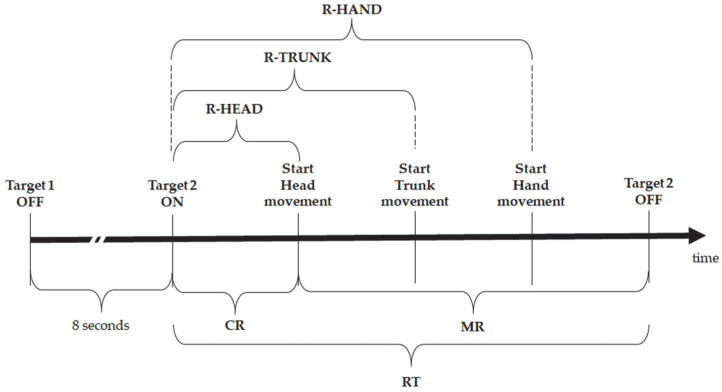
The temporal scheme of the RT’s subintervals.

The RT is divided into time subintervals, calculated through purposely developed MATLAB (Version: 9.13.0—R2022b) code, summarized in Figure 6:

- Cognitive response (CR): time between the appearance of the stimulus and the beginning of the movement of the first anatomical district that reacts;
- Motion response (MR): time between the beginning of the movement of the first anatomical district that reacts and the turn-off of the target. In this phase, the range of motion (ROM) of head rotation, trunk rotation and elbow flexion-extension, and lower limb kinematics were obtained by the dedicated software SMARTAnalyzer (BTS Bioengineering, Milan, Italy). The MR includes the movement of all body districts with sequential involvement of (Figure 6):
  - Head: since the head is the first district of the body to be moved, the response time of the head (hereinafter R-HEAD) coincides with the CR and it is considered as the time between the appearance of the stimulus and the beginning of head rotation;
  - Trunk: the trunk moves after the head, and its response time (hereinafter R-TRUNK) is considered as the time between the appearance of the stimulus and the beginning of trunk rotation;
  - Hand: the hand is used to turn off the target, so it moves last. The hand’s response time (hereinafter R-HAND) is the time between the appearance of the stimulus and the beginning of hand movement. R-HAND may be considered as the real “reaction time” according to the definition commonly used in the literature i.e., the time between the appearance of the stimulus and the beginning of the effective motor act requested to complete the task. In this sense, MR could be considered as a composition of “pre-motor” response, consisting of head and trunk preparatory movements to localize the target, and “motor response” of the hand to reach the target itself.

Consequently, the delay in activation of one part of the body with respect to another can be assessed. Specifically, the delays of the trunk with respect to the head (Delay_1_), of the hand with respect to the trunk (Delay_2_), and of the hand with respect to the head (Delay_3_) can be computed as follows:

(2) $${\mathrm{Delay}}_{1}=R_{\mathrm{TRUNK}}-{\;R}_{\mathrm{HEAD}}$$

(3) $${\mathrm{Delay}}_{2}=R_{\mathrm{HAND}}-{\;R}_{\mathrm{TRUNK}}$$

(4) $${\mathrm{Delay}}_{3}={\;R}_{\mathrm{HAND}}-{\;R}_{\mathrm{HEAD}}={\mathrm{Delay}}_{1}+{\mathrm{Delay}}_{2}$$

### 2.2. Protocol Application

To validate the protocol’s applicability, a pilot study on a group of 10 right-handed healthy subjects (6 females and 4 males, age 25 ± 2 years, height 167.4 ± 8.5 cm) was conducted. The participants underwent the protocol, and the aforementioned temporal and kinematics parameters were calculated during the execution of the four planned tasks.

The study was conducted in accordance with the Declaration of Helsinki and approved by the Ethics Committee of the Politecnico di Milano. All participants were required to sign a written informed consent form, in which the details of the experimental tests were reported.

### 2.3. Protocol Validation

The developed protocol measures RT considering the turn-off as the instant of the minimum distance between the target and the hand. To evaluate the accuracy of the measurements, the RTs recorded by WittySEM and collected on the digital chronometer were compared with the RTs calculated using the protocol for all subjects in each task:

(5) $$\Delta \mathrm{RT}={\mathrm{Mean}\;\mathrm{RT}\;}_{\mathrm{WittySEM}}-\mathrm{Mea}n\;{\mathrm{RT}\;}_{\mathrm{Protocol}}$$

The value of the proximity threshold, selected through WittyManager software (Microgate©, Bolzano, Italy), can be set as “close”, “medium”, or “distant”, and indicates the approaching distance necessary to trigger the proximity sensor. In this study, “medium” distance was selected. However, the proximity sensor is influenced by the background light, and therefore the proximity threshold might vary according to the illumination condition.

For each stimulus, the turn-off distance between the hand’s marker and the target position at the turn-off instant was recorded, and the following analysis has been conducted:

- measurements’ repeatability: to evaluate the intra-variability of the measurements, 50 turn-offs were purposely conducted by the same subject, and the mean and standard deviation were calculated in two different lighting conditions of the laboratory environment (illuminated and dark);
- measurements’ reproducibility: to evaluate the inter-variability of the measurements, the turn-off distance of the tasks conducted by the 10 participants was considered and the related standard deviations were compared.

## 3. Results

### 3.1. Protocol Validation

The comparison between the RTs recorded by WittySEM and the RT calculated using the protocol shows that ΔRT, calculated as the difference between the mean of all RTs, is −0.002 ± 0.014 s.

The evaluation of the measurements’ repeatability shows that, with the same operator, the standard deviations of the turn-off distance values are ±10 mm in dark conditions (laboratory with dark curtains, no artificial light) and ±17 mm with standard (office) illumination (no curtains, office illumination turned on). Results are summarized in Table 1 with a difference between the means of the two conditions equal to 20 mm.

To evaluate the measurements’ reproducibility, the mean and the standard deviations of the turn-off distance for the 10 subjects were calculated. The results are reported in Table 2.

### 3.2. Protocol Application

#### 3.2.1. Temporal Parameters

**Figure 7:**
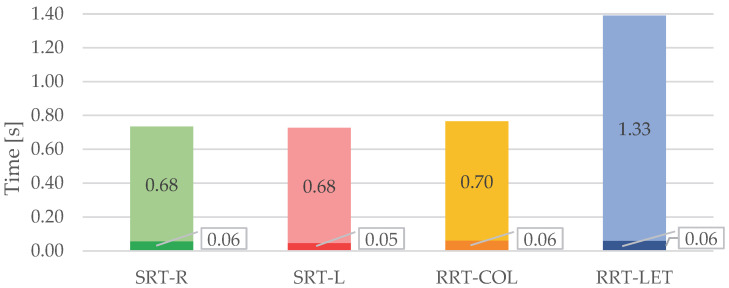
The mean partition of RT in CR and MR (at the bottom and the top of each bar, respectively) for each task.

The temporal parameters summarized in Figure 6 were evaluated by comparing the different tasks and considering the position of the target with respect to the laterality of the hand used to turn it off. The RT values of each task are reported in Table 3 and the relative partition in CR and MR subintervals are represented in Figure 7.

Hereinafter, the simple response time tasks, SRT-R and SRT-L, are depicted in green and red, respectively, while the recognition response time tasks, RRT-COL and RRT-LET, are in yellow and blue, respectively.

In Table 4, the RT values obtained for each task are listed, considering male and female subjects separately.

**Figure 8:**
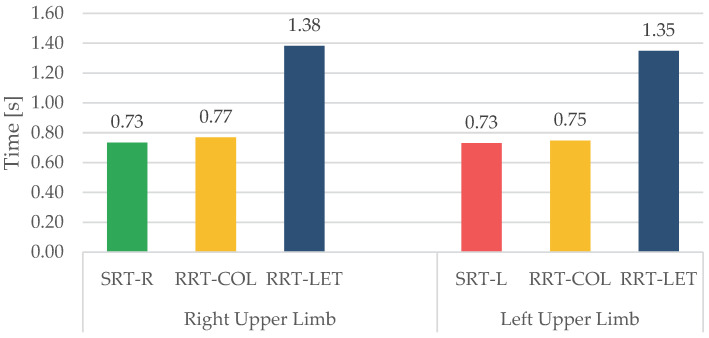
The comparison of mean RTs with the use of the right and left upper limbs for each task.

**Figure 9:**
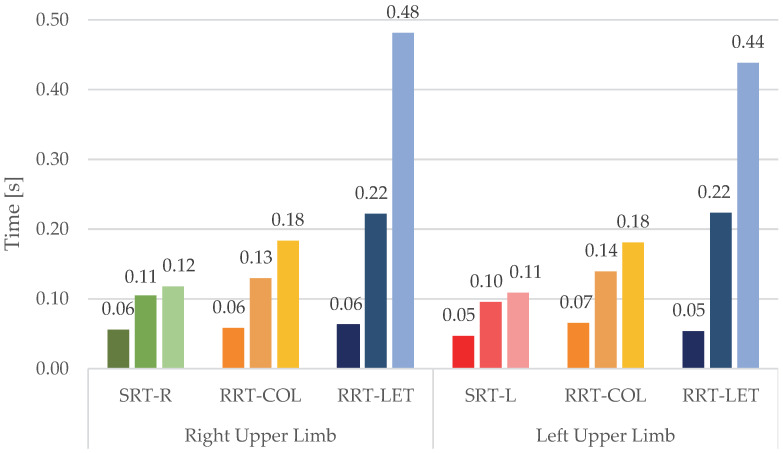
The comparison of mean R-HEAD, R-TRUNK, and R-HAND (from the left bar to the right bar of the grid respectively) for each task with the use of the right and left upper limb.

To compare the response of the right and left upper limbs, we considered separately the use of the two upper limbs during RRT-COL and RRT-LET. We compared the corresponding RTs (Figure 8) R-HEAD, R-TRUNK, and R-HAND (Figure 9) and the delays (Table 5) with those of SRT-R and SRT-L.

**Figure 10:**
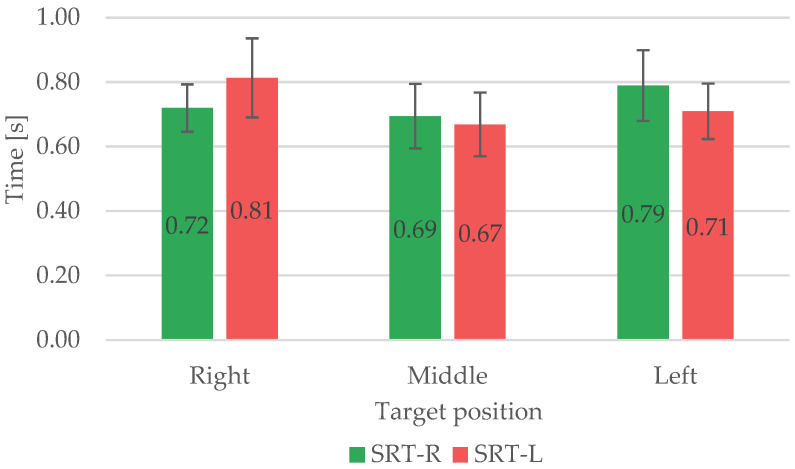
The comparison of RTs for SRT-R and SRT-L tasks according to the position of the target.

To further investigate the differences between the left and right upper limbs, time parameters for SRT tasks were evaluated according to the position of the target on the grid in front of the subject and are reported in Figure 10.

#### 3.2.2. Kinematics

The developed protocol allows for assessing the total-body kinematics. The results show that, for the 10 healthy participants, evaluation of the lower limbs was not necessary since the subjects did not need to move them to turn off the targets.

During the MR intervals, the ROM of elbow flexion-extension and the ROM of head and trunk rotations were calculated for stimuli involving the left and right upper limb according to the target position for each task and reported in Table 6, Table 7 and Table 8.

**Figure 11:**
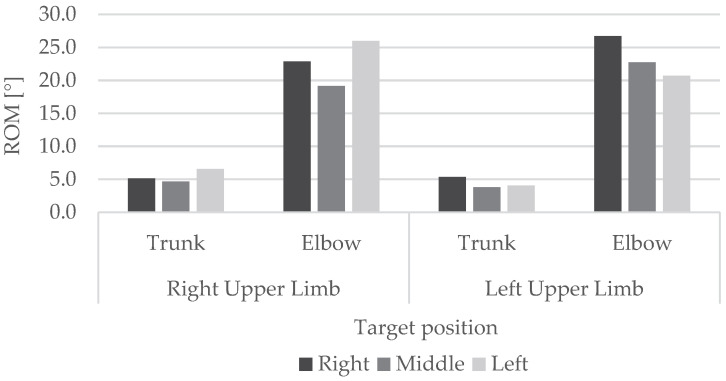
Mean ROM of elbow flexion-extension and mean ROM of trunk rotation ROM considering left and right upper limb separately according to the target position.

Considering all the tasks, the mean of the elbow’s ROM and trunk’s ROM have been calculated and reported in Figure 11, analyzing the left and right upper limbs separately, according to the target position.

## 4. Discussion

This work aimed to develop a new protocol for the simultaneous evaluation of time and motion response during a dual task. The light stimuli are emitted by WittySEM (Microgate©, Bolzano, Italy), a system typically used in sports, composed of eight semaphores. The evaluation of temporal and kinematic parameters is performed through six infrared cameras while the subject is reaching the hand to turn off the target during the execution of tasks of different difficulty levels.

### 4.1. Protocol Validation

To validate the protocol, RTs recorded by WittySEM were compared with those measured considering the position of the hand’s marker: the difference between the means was 0.002 ± 0.014 s. The value is acceptable, considering that, including all the tasks, the measured RTs ranged between 0.51 s and 2.68 s. The evaluation of repeatability, performed on 50 acquisitions purposely made in two different lighting conditions by the same subject, showed that the standard deviations of the turn-off distance are ±10 mm and ±17 mm in dark and illuminated conditions, respectively. Although the intra-variability is minimal compared with the mean thresholds recorded, it may be affected by the light sensitivity of the proximity sensors. Considering the acquisitions made for each of the 10 participants, standard deviations ranging from ±4 mm and ±16 mm confirmed a good intra-variability of the turn-off distance, demonstrating that the subjects stop after turning off the target without physically touching it, and this result may be attributable to the sound signal that is emitted by the system at the instant of task completion. On the other hand, the mean values ranged between ±162 mm and ±226 mm, showing lower reproducibility. In this case, the inter-variability of the measurements may be due to both the different lighting conditions on each day on which the tests were conducted and the different orientations of the hand in approaching the target for each subject.

The results have shown that the method is accurate in RT measuring and that the inter-variability found can be reduced by standardizing lighting conditions.

### 4.2. Protocol Application

A pilot study on a group of 10 right-handed healthy age/height-matched subjects was conducted applying the developed protocol. Temporal and kinematic parameters were measured by comparing the different tasks according to the upper limb used to turn off the target and the location of the target relative to the subject placed in front of the grid.

#### 4.2.1. Temporal Parameters

The RTs were computed for each stimulus as the time taken by the subject to turn off the target. The target position changed during the task, ensuring the independence of the learning effect, also known as the “sequential effect”, which could indeed affect the RT by reducing it [14,22,23].

The mean RT values obtained for each task showed that the response was affected by the task’s difficulty. SRT-R and SRT-L had the same mean value of 0.73 s, which is close to the 0.77 s obtained for RRT-COL. On the other hand, RRT-LET had an RT of almost twice the previous ones. This result confirms what Darbutas et al. [24] and Donders et al. [25] demonstrated in their works: RT worsens in proportion to the increasing complexity of the task. Indeed, we found that distraction generally increases the RT, although colour is strongly helpful in discriminating the target compared with the use of different symbols. Besides, in accordance with other studies [14,26,27,28,29], the mean values of RTs in female subjects were higher than in males, and this difference increases with the task’s difficulty.

The RTs were then divided into different subintervals. The partition in CR and MR shows that the cognitive response, intended as the period of absence of movement after the appearance of the stimulus, is almost equal for the various tasks, indicating that the time required to plan the movement is independent of the type of the stimuli which, on the contrary, does affect the MR. Considering the reaction sequence of the head, trunk, and hand, it is evident that the head begins movement first, to look for the position of the target on the grid. The trunk, and consequently the hand, react with a delay to the head that depends on the task’s difficulty. Specifically, in the RRT-LET tasks, these delays are longer than the corresponding ones in the RRT-COL tasks. In turn, RRT-COL delays are longer than those recorded for SRT tasks. These results confirm that in the RRT-LET task, in which there is no colour help, the “pre-motor phase” is longer because the subject rotates the head and then the trunk for a longer time in search of the target before proceeding to turn it off.

Regarding the laterality of the upper limb used, the results show that, although all the participants were right-handed, there are similar values in RT, R-HEAD, R-TRUNK, and R-HAND comparing the turn-offs done with the left and right limb for all the tasks.

Comparing SRT-R and SRT-L, concerning the target position, the right and left upper limbs have specular behaviour, as expected. The RTs are lower in the case of the middle position than in the case of the lateral position, independently of the upper limb used. On the other hand, when the target is on the lateral pole of the grid, the RTs are slightly higher (some ms) when the limb used is contralateral to the target position and vice versa if it is ipsilateral. This behaviour is the same for SRT-L and SRT-R.

The results show that, when the subject can choose the upper limb, there is a tendency to use the preferred laterality. To optimize the execution and compensate for the difficulty of the tasks, the subject preferred the right upper limb. This is demonstrated by the absence of the use of the left limb to turn off the semaphores on the right pole during RRT-LET and RRT-COL. But, despite this preference, the RTs would not be affected by laterality. These findings are independent of the task’s difficulty [30].

#### 4.2.2. Kinematics

For all the tasks, the total body kinematic parameters were calculated during the MR subintervals. No foot or lower limb movements were analysed as the subjects were able to reach the target with only upper body movement.

The ROM results highlighted that there is a large variability in angle values with high standard deviations. This can be associated with the different movements performed by the subjects. In addition, the vertical position of the target, not considered in this protocol, can also influence the angular values. The ROM analysis shows that there is a similar behaviour between the left and right upper limbs: as expected, the ROM is higher when the target is in a contralateral position with respect to the upper limb used to turn off the target.

## 5. Conclusions

In the present study, a protocol for the simultaneous assessment of time and motion response during different difficulty levels of dual tasks by using WittySEM and an optoelectronic system was developed. The protocol was applied and validated in an exploratory pilot study on a small healthy population. The results confirmed the dependency of the response time on the task’s difficulty, while also providing information about kinematics.

With a perspective to apply the protocol in a clinical study to characterize a larger pediatric population, the proposed method overcomes the limits of the commonly adopted protocols: since the analysis of the response is based on the use of an optoelectronic system (and WittySEM is only used to generate visual stimuli), the test is extremely versatile, and results are accurate and independent from the experimental setup, allowing comparison between tests performed in different laboratories. The method may be adapted and applied with other analogous instruments generating different kinds of stimuli, and the playful nature of the tasks makes the protocol suitable for pediatric context. It also seems possible to reasonably vary the tasks to adjust the method to specific pathologies.

Another advantage is the motion analysis that provides a more comprehensive assessment of the response in terms of kinematics also. The evaluation of the total-body kinematics may be redundant on healthy subjects with this specific experimental setup, but it is expected to provide meaningful information in the case of subjects with deficits, for example in detecting compensatory motor strategies. In this sense, quantitative measures of the response may also circumvent the low sensitivity of the clinical scales in view of quantification of motor and cognitive impairments.

Despite the promising results in this pilot study, further work to test the protocol applicability to a pathological pediatric population, and an in-depth analysis to characterize the relative control group, needs to be conducted.

## Figures and Tables

**Table 1:** Mean and standard deviation of the turn-off distance in dark and illuminated conditions for the same operator.

Lighting Condition	Mean [mm]	Standard Deviation [mm]
Dark	122	±10
Illuminated	102	±17

**Table 2:** Mean and standard deviation of the turn-off distance for each subject.

Subject	1	2	3	4	5	6	7	8	9	10	Population
Mean [mm]	217	226	175	191	194	210	204	203	162	211	199
Standard deviation [mm]	±7	±7	±12	±15	±5	±11	±4	±11	±16	±10	±28

**Table 3:** Mean and standard deviation of the RTs for each task.

	**SRT-R**	**SRT-L**	**RRT-COL**	**RRT-LET**
Mean [s]	0.73	0.73	0.77	1.40
Standard deviation [s]	±0.02	±0.03	±0.02	±0.17

**Table 4:** Mean and standard deviation of the RTs for each task considering female and male subjects.

	SRT-R [s]	SRT-L [s]	RRT-COL [s]	RRT-LET [s]
Male	0.72 ± 0.09	0.71 ± 0.08	0.75 ± 0.11	1.34 ± 0.50
Female	0.75 ± 0.10	0.73 ± 0.11	0.77 ± 0.10	1.42 ± 0.44

**Table 5:** Mean temporal delays for each task with the use of the right and left upper limb.

	Right Upper Limb			Left Upper Limb		
	SRT-R	RRT-COL	RRT-LET	SRT-L	RRT-COL	RRT-LET
${Delay}_{1}$ [s]	0.05	0.07	0.16	0.05	0.07	0.17
${Delay}_{2}$ [s]	0.01	0.05	0.26	0.01	0.04	0.22
${Delay}_{3}$ [s]	0.06	0.12	0.42	0.06	0.11	0.39

**Table 6:** Mean and standard deviation of ROM of elbow flexion-extension for all tasks considering left and right upper limb separately according to the target position.

	Right Upper Limb			Left Upper Limb		
Target Position	SRT-R[°]	RRT-COL[°]	RRT-LET[°]	SRT-L[°]	RRT-COL[°]	RRT-LET[°]
Right	23.6 ± 12	26.0 ± 17	19.0 ± 15	26.7 ± 10	-	-
Middle	15.4 ± 8	17.9 ± 13	24.2 ± 12	27.2 ± 12	23.7 ± 10	17.4 ± 6
Left	21.5 ± 12	26.6 ± 17	29.9 ± 7	20.5 ± 12	22.5 ± 17	19.1 ± 14

**Table 7:** Mean and standard deviation of ROM of head rotation for all tasks considering left and right upper limb separately according to the target position.

	Right Upper Limb			Left Upper Limb		
Target Position	SRT-R[°]	RRT-COL[°]	RRT-LET[°]	SRT-L[°]	RRT-COL[°]	RRT-LET[°]
Right	11.8 ± 6	20.3 ± 7	20.8 ± 11	20.0 ± 9	-	-
Middle	12.8 ± 8	13.7 ± 6	13.7 ± 6	14.0 ± 6	16.9 ± 7	19.1 ± 14
Left	21.2 ± 7	18.4 ± 5	30.7 ± 13	16.1 ± 9	17.3 ± 6	22.8 ± 9

**Table 8:** Mean and standard deviation of ROM of trunk rotation for all tasks considering left and right upper limb separately according to the target position.

	Right Upper Limb			Left Upper Limb		
Target Position	SRT-R[°]	RRT-COL[°]	RRT-LET[°]	SRT-L[°]	RRT-COL[°]	RRT-LET[°]
Right	4.5 ± 3	5.4 ± 3	5.5 ± 3	5.3 ± 4	-	-
Middle	3.8 ± 2	5.1 ± 2	5.0 ± 3	6.3 ± 5	3.2 ± 2	1.9 ± 1
Left	6.2 ± 3	5.6 ± 4	7.9 ± 4	4.8 ± 4	3.7 ± 2	3.7 ± 3

## Data Availability

Data available on request due to restrictions, e.g., privacy or ethical.
